# ALYREF regulates glycolysis and promotes the progression of endometrial cancer through m^5^C modification of PKM2 mRNA

**DOI:** 10.3389/fcell.2026.1831562

**Published:** 2026-10-02

**Authors:** Yun Feng, Xueyin Li

**Affiliations:** 1 Department of Gynaecology, The First Affiliated Hospital of Zhengzhou University, Zhengzhou, Henan, China; 2 Department of Urology, The First People’s Hospital of Zhengzhou, Zhengzhou, Henan, China

**Keywords:** ALYREF, endometrial cancer, glycolysis, m5C modification, PKM2, proliferation

## Abstract

Endometrial cancer (EC) is one of the most common gynecologic malignancies, and effective treatment options for advanced or recurrent cases remain limited. Here, we investigated the biological function and molecular mechanism of the RNA-binding protein ALYREF in EC. Bioinformatics analysis of public datasets revealed that ALYREF is significantly upregulated in EC tissues, and high ALYREF expression is associated with poor overall survival in the Kaplan-Meier Plotter cohort. Clinical validation using paired EC tissues confirmed ALYREF upregulation at protein level, and high ALYREF expression was associated with aggressive clinicopathological features. Functional assays revealed that ALYREF promotes EC cell proliferation, migration, and invasion *in vitro* and enhances tumor growth and metastasis *in vivo*. Mechanistically, ALYREF increases the stability of PKM2 mRNA, which we demonstrate to be m^5^C-modified in EC, leading to elevated PKM2 protein expression, enhanced glycolytic metabolism and tumor progression. Rescue experiments confirmed that PKM2 is an important downstream effector of ALYREF-mediated oncogenic activity. Collectively, this study identifies the ALYREF-PKM2 axis as an important regulator linking RNA methylation and glucose metabolism in EC, suggesting that this axis may be a potential therapeutic target for EC.

## Background

Endometrial cancer (EC) is one of the most common gynecological malignancies, and its global incidence has continued to rise in recent decades, posing a substantial burden to women’s health worldwide (Crosbie et al., 2022). Despite improvements in surgical techniques, radiotherapy, and targeted therapy, effective treatment options for patients with advanced or recurrent EC remain limited, and the prognosis of these cases is still unsatisfactory (Corr et al., 2025). The pathogenesis of EC is complex and involves multiple signaling pathways and epigenetic regulatory layers, including RNA modifications (He et al., 2024), as well as aberrant activation of various oncogenic signaling cascades (Corr et al., 2025; Miao et al., 2025). Emerging therapeutic approaches, including novel targeted drug conjugates, are also under preclinical investigation for this malignancy (Markatos et al., 2024). Nevertheless, further identification of novel molecular biomarkers and therapeutic targets remains critical to improve clinical management and outcomes for patients with EC.

RNA modifications have emerged as critical regulators of cancer metabolism and tumor progression (Yang et al., 2026; Zhang et al., 2025; Shi et al., 2026). 5-Methylcytosine (m^5^C) is a prevalent RNA modification initially discovered in tRNA and rRNA and subsequently identified in mRNA (Lu et al., 2024; Squires et al., 2012; Khoddami and Cairns, 2013). This modification can modulate RNA stability, export, and translation efficiency, thereby exerting widespread regulatory effects on gene expression and cellular function (Roundtree et al., 2017). The dynamic m^5^C landscape is orchestrated by a coordinated set of methyltransferases (writers), demethylases (erasers), and reader proteins (readers). Dysregulation of these components leads to aberrant m^5^C modification, which can activate oncogenes or silence tumor suppressor genes, thereby driving tumorigenesis and cancer progression (Zhang et al., 2025; Sun et al., 2020; Chen et al., 2019). Although accumulating evidence has revealed critical roles of m^5^C in various cancers (Zhang et al., 2025; Sun et al., 2020; Chen et al., 2019), its specific function and molecular mechanisms in endometrial cancer remain largely unexplored, even as RNA epitranscriptomic alterations have been increasingly recognized across gynecologic malignancies (He et al., 2024). Recent comprehensive analyses have identified m^5^C dysregulation in female cancers as a critical post-transcriptional regulatory layer (Yang et al., 2026). In EC, the m^5^C writer enzyme NSUN2 is upregulated and predicts poor prognosis (Yang et al., 2023), and promotes malignant phenotypes through m^5^C modification of SLC7A11 mRNA, which is recognized by the reader YBX1 to enhance transcript stability and confers ferroptosis resistance (Chen et al., 2024). While the NSUN2-YBX1 writer-reader axis has been partially characterized, how other m^5^C readers decode and execute the oncogenic programs initiated by aberrant m^5^C writers in EC remains poorly defined.

Within the m^5^C regulatory network, RNA “reader” proteins serve as the crucial molecular bridge linking RNA methylation with post-transcriptional gene regulation. Among these readers, Aly/REF export factor (ALYREF) is a well-characterized RNA-binding protein (RBP) that functions as a key m^5^C reader, recognizing methylated mRNAs and facilitating their maturation, nuclear export, and stability (Pacheco-Fiallos et al., 2023; Yang et al., 2017). Notably, ALYREF is highly expressed in multiple cancer types, where it regulates cell proliferation, migration, and metabolism by binding m^5^C-modified transcripts (Xue et al., 2023; Jin et al., 2024). Recently, ALYREF was shown to recognize m^5^C-modified BIRC5 mRNA and enhance its stability, thereby promoting ovarian cancer progression and associated glycolytic phenotypes (Tian et al., 2025), establishing a direct precedent for ALYREF-driven oncogenic mechanisms in gynecological malignancies. Nevertheless, whether ALYREF directly targets metabolic enzymes such as PKM2 via m^5^C-associated mRNA stabilization to drive glycolysis in endometrial cancer has not been explored.

In this study, we systematically investigated the biological role and molecular mechanism of ALYREF in EC. We demonstrated that ALYREF is significantly overexpressed in EC tissues and cell lines and is associated with unfavorable patient outcomes. Mechanistically, ALYREF increases the stability of PKM2 mRNA, which we demonstrate to be m^5^C-modified in EC, thereby promoting glycolytic metabolism and tumor progression. This work characterizes an ALYREF–PKM2 regulatory axis that connects RNA methylation and metabolic reprogramming in EC and provides potential therapeutic insights for metabolic intervention.

## Materials and methods

### Clinical tissue samples

A total of 50 paired EC tumor and adjacent normal endometrial tissues were collected from patients undergoing surgical resection at the Department of Pathology, The First Affiliated Hospital of Zhengzhou University. Among these, three representative pairs were randomly selected for preliminary Western blot and RT-qPCR validation using snap-frozen tissues, while all 50 pairs were subjected to immunohistochemical (IHC) analysis. Adjacent normal tissues served as the normal control group for both experiments. Clinicopathological information, including age, FIGO stage, histological grade, myometrial invasion depth, and lymph node metastasis status, was retrospectively retrieved from electronic medical records. All patients provided written informed consent, and the study was approved by the Institutional Ethics Committee (approval number:2025-KY-0286) on 10 May 2025. The cohort included patients with complete clinicopathological records, and the study commenced after ethical approval.

### Cell lines and culture

Human endometrial cancer cell lines (Ishikawa, HEC-1-A, and AN3CA) were obtained from the Shanghai Institute of Cell Biology, Chinese Academy of Sciences. The normal endometrial epithelial cell line EEC, which served as the non-tumour control, was purchased from the American Type Culture Collection (ATCC). All cells were cultured in Dulbecco’s Modified Eagle’s Medium (DMEM; Corning, 10–013-CVRC) supplemented with 10% fetal bovine serum (FBS; Dakewe, C04001) and 1% penicillin/streptomycin (Solarbio, P1400) at 37 °C in a humidified incubator containing 5% CO_2_.

### Plasmid and shRNA transfection

Transient transfections were performed using Lipofectamine 3,000 (Invitrogen) according to the manufacturer’s instructions. ALYREF and PKM2 overexpression plasmids were synthesized by Suzhou Kingbio Biotechnology Co., Ltd. Company, with empty vector (Vector) used as the negative control for overexpression experiments. Short hairpin RNA (shRNA) sequences targeting ALYREF were listed in the Supplementary Table S1, with a scramble shRNA (sh-NC) used as the negative control for knockdown experiments. Cells were harvested 48 h post-transfection for subsequent assays.

### Western blot

Total proteins from cells or tissues were extracted using RIPA lysis buffer supplemented with PMSF (1 mM) on ice for 30 min, and quantified by the BCA method. Equal amounts of protein were separated by SDS-PAGE and transferred onto PVDF membranes, followed by blocking with 5% nonfat milk. Membranes were incubated overnight at 4 °C with primary antibodies and then with HRP-conjugated secondary antibodies for 1 h at room temperature. Detection was performed using an ECL substrate. Band intensities were quantified using ImageJ software, and target protein levels were normalized to β-actin. Primary antibodies included ALYREF (Proteintech, 16690-1-AP), PKM2 (Proteintech, 15822-1-AP), E-cadherin (Proteintech, 20874-1-AP), N-cadherin (Proteintech, 22018-1-AP), Vimentin (Proteintech, 10366-1-AP), MMP2 (Proteintech, 10373-2-AP), and β-actin (Proteintech, 66009-1-Ig).

### Quantitative reverse transcription PCR (RT-qPCR)

Total RNA was extracted using TriQuick Reagent (Solarbio, R1100). Reverse transcription was performed with the HiScript II Q RT SuperMix for qPCR kit (Vazyme, R223). Quantitative PCR was carried out using SYBR Green Master Mix (Solarbio, SY1020) on a StepOnePlus system (Applied Biosystems). β-Actin was used as an internal reference, and relative gene expression levels were calculated by the 2^–ΔΔCt^ method. Primer sequences are listed as follows:

**Table:** 

Name	Primer sequences
h-ALYREF-F	GGC​AGC​ACG​ATC​TTT​TCG​AC
h-ALYREF-R	CTG​TTC​CTA​AGC​TGC​GAC​CA
h-actin-F	GAC​AGC​CAT​GGA​GGA​CGA​G
h-actin-R	CAA​GTC​ACA​GCG​AAC​TTG​CC
h-GAPDH-F	TCA​AGG​CTG​AGA​ACG​GGA​AG
h-GAPDH-R	TCG​CCC​CAC​TTG​ATT​TTG​GA
h-PKM2-F	AGA​AAG​GTG​CCG​ACT​TCC​TG
h-PKM2-R	GCT​CGA​CCC​CAA​ACT​TCA​GA

### Immunohistochemistry (IHC)

Formalin-fixed, paraffin-embedded tissue samples were sectioned at 5 µm. After deparaffinization, rehydration, and antigen retrieval using citrate or EDTA buffer, slides were blocked with 5% goat serum and incubated overnight at 4 °C with primary antibodies against ALYREF (Proteintech, 16690-1-AP, 1:500), PKM2 (Proteintech, 15822-1-AP, 1:600), Ki-67 (Proteintech, 27309-1-AP, 1:800), E-cadherin (Proteintech, 20874-1-AP, 1:5000), and Vimentin (Affinity, AF7013, 1:300). Sections were incubated with HRP-conjugated secondary antibodies at 37 °C for 1 h, developed with DAB, and counterstained with hematoxylin. Images were captured using a 3DHISTECH viewer, and H-score quantification was performed independently by two investigators in a blinded manner using ImageJ IHC Profiler plugin.

### Cell counting Kit-8 (CCK-8) and colony formation assays

For proliferation assays, cells were seeded into 96-well or 6-well plates. Cell viability was measured using the CCK-8 kit (CK04, Dojindo) according to the manufacturer’s instructions. For colony formation, cells were cultured for 10–14 days, fixed with paraformaldehyde, stained with 0.5% crystal violet, and manually counted.

### Transwell migration and invasion assays

Cell migration and invasion were assessed using Transwell chambers (Corning). For invasion assays, inserts were pre-coated with Matrigel (BD Biosciences). After incubation for 24–48 h, cells on the lower membrane surface were fixed, stained with crystal violet, and counted under a microscope.

### Animal experiments

Female BALB/c nude mice (4–6 weeks old) were purchased from the SPF(BEIJING) BIOTECHNOLOGY Animal Center (License Number: SCXK (JING)2024–0001). For subcutaneous xenograft models, Ishikawa or sh-ALYREF Ishikawa cells (5 × 10^6^ cells/mouse) were injected subcutaneously into the flanks of mice (5 mice per group). Tumor volumes and body weights were measured every 3 days using calipers and a digital scale, respectively. Tumor volumes were calculated as length × width^2^ × 0.5. The humane endpoint criteria were predefined as tumor diameter ≥15 mm, tumor volume ≥1500 mm^3^, >20% body weight loss, or the experiment reaching day 21, whichever came first; mice meeting any criterion were immediately euthanized. For the metastasis model, cells were injected via the tail vein to establish hematogenous dissemination (5 mice per group). Tumor tissues and major organs were collected for hematoxylin–eosin (HE) staining and immunohistochemistry (IHC) analyses. All animal procedures were approved by the Institutional Animal Care and Use Committee (Approval No. ZZU-LAC20251226, approved on 27 April 2025) and were performed commencing in June 2025.

### Dot blot and m^5^C RNA immunoprecipitation (m^5^C-RIP) assays

Dot blot analysis was performed to assess the global m^5^C modification levels. Equal amounts of total RNA were spotted onto nylon membranes, UV-crosslinked, and hybridized with an anti-m^5^C antibody, followed by HRP-conjugated secondary antibody detection.

For m^5^C-RIP assays, total RNA was incubated with anti-m^5^C antibody (Proteintech, 68301-1-Ig) to immunoprecipitate m^5^C-modified transcripts. The enriched RNA was purified and analyzed by RT-qPCR to quantify PKM2 mRNA enrichment in the m^5^C-immunoprecipitated fraction.

### Actinomycin D transcriptional inhibition assay

Cells were treated with Actinomycin D (5 μg/mL; Sigma-Aldrich) to inhibit transcription. Total RNA was collected at 0, 2, 4, 6, and 8 h after treatment, and PKM2 mRNA levels were measured by RT-qPCR to evaluate transcript stability. The relative half-life of PKM2 mRNA was calculated by normalizing to the initial time point.

### Public database analysis

Gene Expression Profiling Interactive Analysis (GEPIA, http://gepia.cancer-pku.cn/) was used to examine ALYREF expression and the gene-level association between ALYREF and PKM. GEPIA uses TCGA and GTEx RNA-seq data recomputed from raw sequencing data by the UCSC Xena project through a uniform processing pipeline, thereby mitigating computational batch effects attributable to differences in alignment and expression quantification. For the correlation analysis, Gene A was set to ALYREF and Gene B to PKM; UCEC tumour, UCEC normal, and GTEx uterus datasets were selected; Spearman’s rank correlation was used; and the optional gene-normalization field was left blank. Correlations were calculated using the non-log expression scale and displayed on log2 (TPM) axes. The query was defined at the PKM gene level.

Overall survival according to ALYREF expression was examined using the archived UCEC output from the pan-cancer RNA-sequencing service of Kaplan–Meier Plotter (https://kmplot.com/analysis/index.php?p=service&cancer=pancancer_rnaseq). According to the published processing workflow for this resource, TCGA RNA-seq HTSeq counts were normalized using DESeq and subsequently rescaled within each sample to reduce batch effects. The archived output included 422 patients (ALYREF-low, n = 223; ALYREF-high, n = 199), and patient grouping and survival statistics were generated by the platform.

### TCGA-UCEC RNA-sequencing preprocessing and gene set enrichment analysis

Gene-level TCGA uterine corpus endometrial carcinoma (TCGA-UCEC) expression data were obtained from the GDC UCSC Xena hub (TCGA-UCEC.star_counts.tsv; accessed 20 August 2026; GENCODE v36 annotation). Samples were restricted to primary tumours using TCGA sample-type code 01, yielding 549 primary-tumour columns. When multiple primary-tumour samples were available for one patient, the lexicographically first sample barcode was retained, yielding 545 unique patients. Xena log2 (x + 1) values were returned to the STAR unstranded count scale by exact inverse transformation without rounding.

The analysis was restricted to protein-coding genes. Genes were retained when counts per million (CPM) exceeded 1 in at least 20% of primary tumours, corresponding to 109 of 545 samples. Library sizes were normalized using the trimmed mean of M-values method, and log2 CPM values were calculated with a prior count of 1 using edgeR version 4.2.2.

For preranked GSEA, protein-coding genes were ordered by their Spearman correlation coefficients with ALYREF across the 545 primary tumours. Duplicate gene symbols were resolved by retaining the entry with the largest absolute correlation coefficient, with mean expression and gene symbol used as secondary ordering variables. After excluding ALYREF itself, 14,867 unique gene symbols were ranked. All 50 Hallmark gene sets from MSigDB 2025.1. Hs were obtained using msigdbr version 25.1.1. Enrichment was tested using fgsea version 1.30.0 with minSize = 10, maxSize = 500, and eps = 0. P values were adjusted across all tested Hallmark pathways using the Benjamini–Hochberg method.

### Statistical analysis

All quantitative data are presented as mean ± standard deviation (SD). Statistical analyses were performed using GraphPad Prism version 8.0 (GraphPad Software, San Diego, CA, United States). Comparisons between two independent groups were conducted using two-tailed Student’s *t*-tests. Comparisons between paired clinical tissues were analyzed using paired *t*-test. For multiple-group comparisons, one-way analysis of variance (ANOVA) was performed, followed by Tukey’s multiple comparisons test for pairwise comparisons. For xenograft tumor growth curves, two-way repeated-measures ANOVA was performed with group and time as factors, followed by Bonferroni *post hoc* test. Correlation between ALYREF and PKM2 H-scores was assessed by Spearman correlation analysis, as the data were not normally distributed (Shapiro-Wilk, *p* < 0.05). Associations between protein expression and clinicopathological parameters were analyzed using *χ* ^2^ test. A *p*-value <0.05 was considered statistically significant.

## Results

### ALYREF is upregulated in endometrial cancer and associated with aggressive clinicopathological features

To explore the potential role of ALYREF in endometrial cancer, we first examined its expression using GEPIA. ALYREF expression was higher in EC tissues than in normal endometrial tissues (Figure 1A). In the archived Kaplan–Meier Plotter UCEC output, higher ALYREF expression was associated with shorter overall survival (n = 422; ALYREF-low, n = 223; ALYREF-high, n = 199; HR = 1.87, 95% CI 1.09–3.20; log-rank *p* = 0.021; Figure 1B).

**FIGURE 1:**
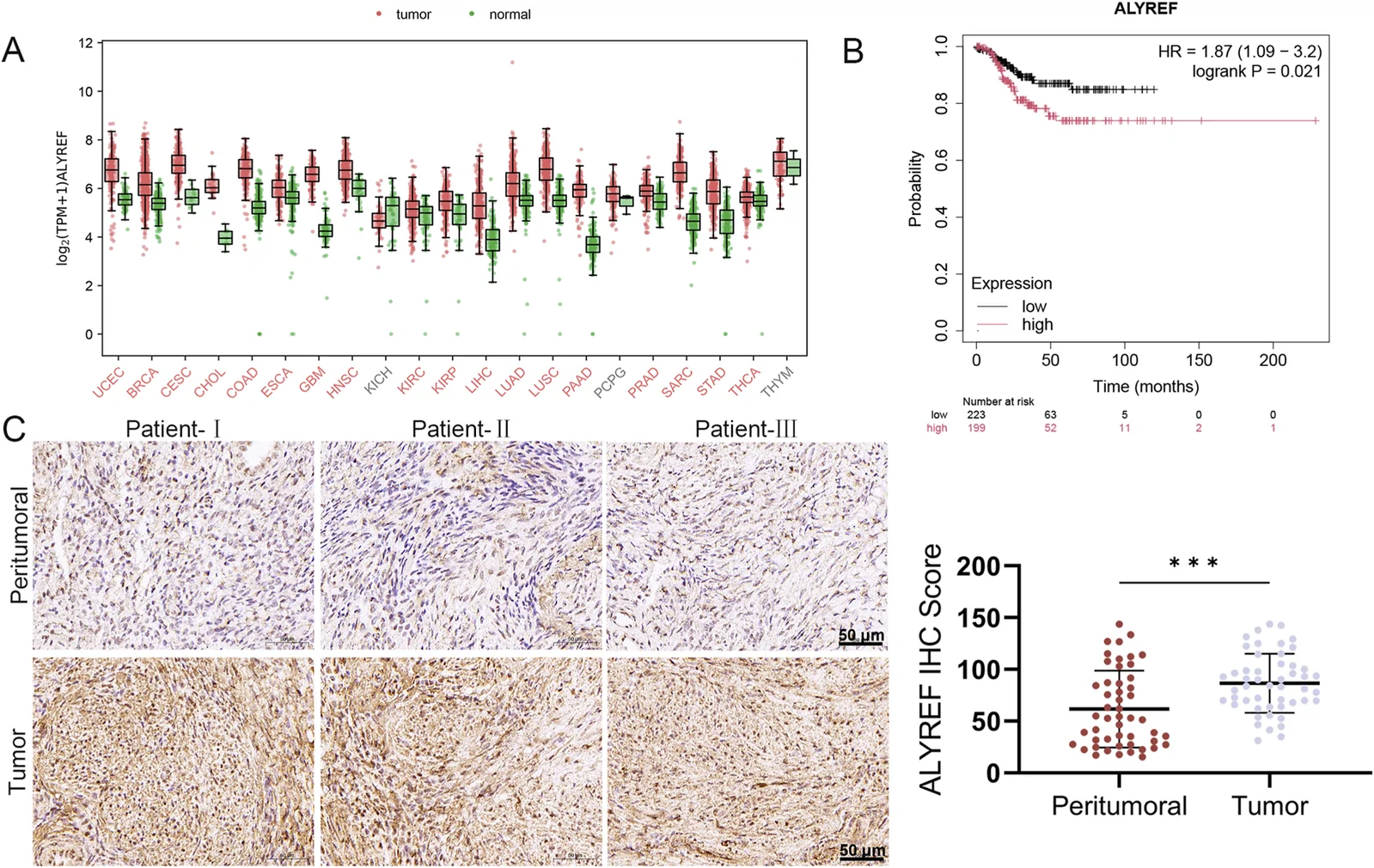
ALYREF is upregulated in endometrial cancer and associated with shorter overall survival. **(A)** Expression profiles of ALYREF in endometrial cancer and normal tissues from GEPIA (TCGA and GTEx). **(B)** Kaplan–Meier Plotter analysis showing the association between ALYREF expression and overall survival in the archived UCEC output (n = 422; ALYREF-low, n = 223; ALYREF-high, n = 199; HR = 1.87, 95% CI 1.09–3.20; log-rank *p* = 0.021). **(C)** Representative immunohistochemistry (IHC) staining of ALYREF in paired peritumoral and tumor tissues from our clinical cohort (50 pairs, representative images from three patients). Quantitative IHC scores were derived from all 50 paired cases (paired *t*-test). Scale bar = 50 µm. Data are presented as mean ± SD; *p < 0.05, **p < 0.01, ***p < 0.001.

We next validated ALYREF expression in clinical specimens. Western blot analysis of three paired EC and adjacent normal tissues confirmed a significant increase in ALYREF protein levels in tumor tissues (*p* < 0.001; Supplementary Figure S1). To extend this validation to an expanded cohort, IHC staining was subsequently performed on 50 paired EC and adjacent normal tissues. ALYREF was predominantly localized in the nucleus and exhibited markedly stronger staining intensity in tumor tissues compared with adjacent noncancerous tissues (*p* < 0.001, Figure 1C). Moreover, high ALYREF expression was significantly associated with advanced FIGO stage, higher histological grade, deeper myometrial invasion, lymph node metastasis, and shorter survival (<5 years), whereas no significant correlations were observed with age (Table 1). Together, these data indicate that ALYREF is upregulated in EC and correlates with aggressive clinicopathological features in our clinical cohort, and that the independent public cohort supports its prognostic association with overall survival.

**TABLE 1:** Correlation between ALYREF expression and clinicopathological characteristics of endometrial cancer patients.

Characteristic	Category	ALYREF low (n = 25)	ALYREF high (n = 25)	*χ* ^2^	*P* value
Age (years)	<60	20 (80.00%)	19 (76.00%)	0.117	0.733
​	≥60	5 (20.00%)	6 (24.00%)	​	​
FIGO stage	I-II	18 (72.00%)	11 (44.00%)	4.023	0.045
​	III-IV	7 (28.00%)	14 (56.00%)	​	​
Histological grade	G1	12 (48.00%)	4 (16.00%)	10.278	0.006
​	G2	11 (44.00%)	10 (40.00%)	​	​
​	G3	2 (8.00%)	11 (44.00%)	​	​
Myometrial invasion	<1/2	18 (72.00%)	9 (36.00%)	6.522	0.011
​	≥1/2	7 (28.00%)	16 (64.00%)	​	​
Lymph node metastasis	No	23 (92.00%)	14 (56.00%)	8.420	0.004
​	Yes	2 (8.00%)	11 (44.00%)	​	​
Survival	≥5 years	19 (76.00%)	12 (48.00%)	4.160	0.041
​	<5 years	6 (24.00%)	13 (52.00%)	​	​

Data are presented as n (%). Comparisons were performed using *χ* ^2^ test. *P* < 0.05 was considered statistically significant.

### ALYREF promotes proliferation, migration, and invasion in endometrial cancer cells

We next examined ALYREF expression in the normal endometrial epithelial cell line EEC and three EC cell lines. ALYREF was upregulated in all three cancer cell lines relative to the normal control (Figure 2A). Based on this endogenous expression profile, we performed loss-of-function and gain-of-function assays in different cell lines, selecting high-expressing cells for knockdown and low-expressing cells for overexpression (Ye et al., 2026; Xie et al., 2025): ALYREF was knocked down in Ishikawa cells (highest endogenous expression among EC lines) and overexpressed in HEC-1-A cells (lower endogenous expression), with both efficiencies confirmed by Western blot and RT-qPCR (Figure 2B).

**FIGURE 2:**
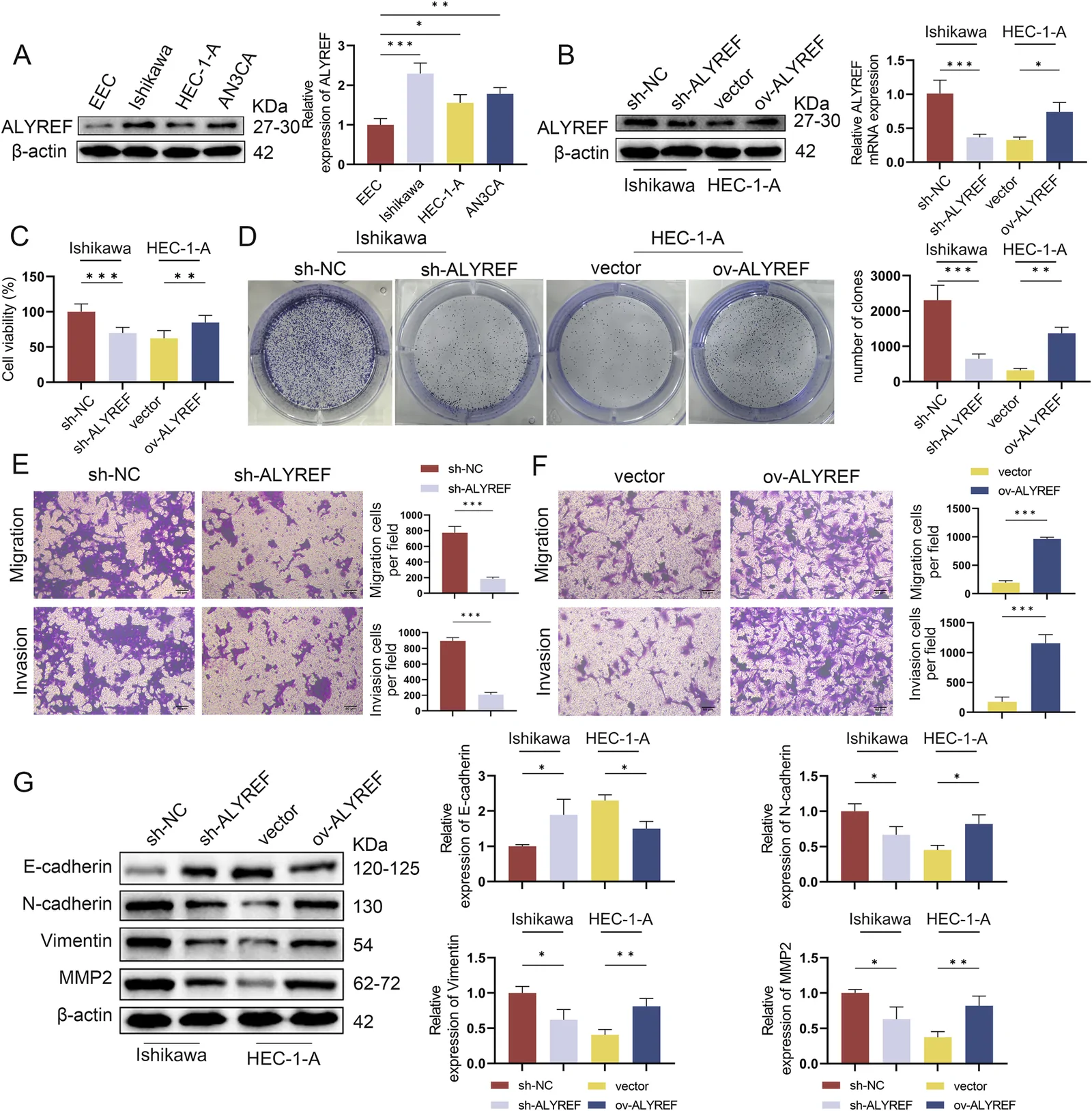
ALYREF promotes proliferation, migration, and invasion in endometrial cancer cells. **(A)** Western blot analysis of ALYREF protein expression in normal endometrial cell line (EEC) and EC cell lines (Ishikawa, HEC-1-A, AN3CA). **(B)** Validation of ALYREF knockdown and overexpression by Western blot and RT-qPCR. **(C)** CCK-8 assay showing cell viability after ALYREF modulation. **(D)** Colony formation assays evaluating the proliferative ability of Ishikawa and HEC-1-A cells with altered ALYREF expression. **(E,F)** Transwell migration and invasion assays in Ishikawa **(E)** and HEC-1-A **(F)** cells with ALYREF knockdown or overexpression; Scale bar: 50 μm. **(G)** Western blot and quantification of EMT-related markers (E-cadherin, N-cadherin, Vimentin, MMP2). sh-NC: scramble shRNA negative control for knockdown; Vector: empty vector control for overexpression. Data are presented as mean ± SD from three independent biological replicates; two-tailed unpaired *t*-test; *p < 0.05, **p < 0.01, ***p < 0.001.

Functional assays demonstrated that ALYREF knockdown markedly suppressed cell proliferation, whereas ALYREF overexpression promoted proliferation as shown by CCK-8 and colony formation assays (Figures 2C,D). Transwell assays further revealed that ALYREF depletion significantly inhibited cell migration and invasion, while ALYREF overexpression enhanced these capacities (Figures 2E,F). Given that ALYREF significantly altered cell migration and invasion, we assessed canonical epithelial–mesenchymal transition (EMT) markers, as EMT is the principal mechanism driving these invasive phenotypes in epithelial cancers (Aiello and Kang, 2019). Western blot analysis showed that ALYREF knockdown increased E-cadherin expression but decreased N-cadherin, Vimentin, and MMP2 levels, indicating a reversal of EMT. Opposite trends were observed following ALYREF overexpression (Figure 2G).

### ALYREF enhances tumor growth and metastasis *in vivo*

To verify the oncogenic role of ALYREF *in vivo*, subcutaneous xenograft and tail-vein metastasis models were established in nude mice. Tumor growth was significantly suppressed in mice injected with ALYREF-knockdown Ishikawa cells compared with control cells, as indicated by reduced tumor volume (*p* < 0.05) and weight (*p* < 0.001, Figures 3A–C). HE staining showed that tumors in the ALYREF-knockdown group exhibited more regular cell morphology and decreased nuclear atypia (Figure 3D). IHC staining confirmed markedly reduced expression of ALYREF and proliferation marker Ki-67 in xenografts from the knockdown group (both *p* < 0.001; Figure 3E).

**FIGURE 3:**
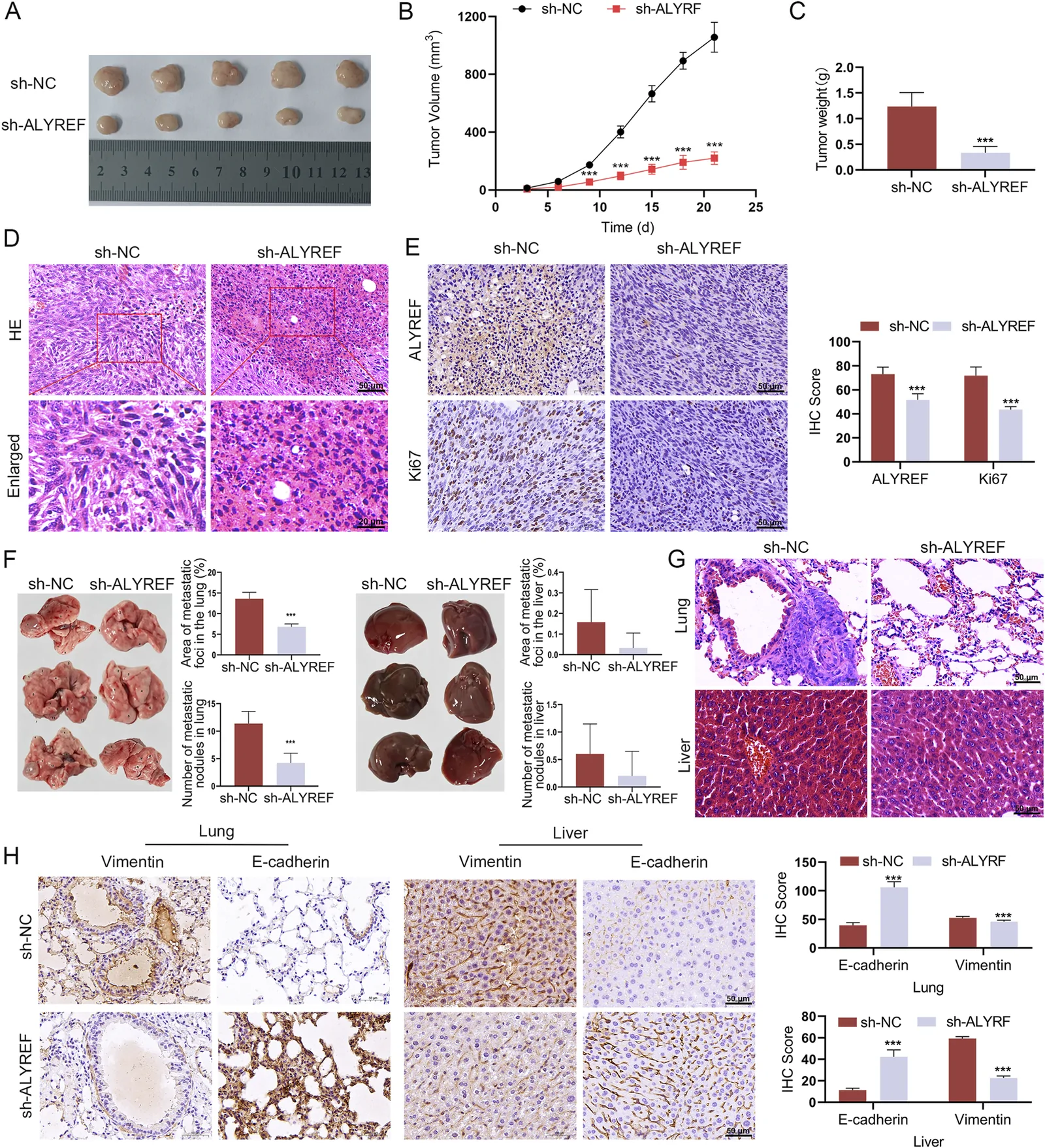
ALYREF promotes tumor growth *in vivo* and distant metastasis in endometrial cancer. **(A)** Representative images of xenograft tumors derived from Ishikawa cells with or without ALYREF knockdown. **(B)** Tumor volume growth curves over time. **(C)** Tumor weight at endpoint. **(D)** Hematoxylin-eosin (HE) staining of xenograft tumors. **(E)** IHC staining and quantification of ALYREF and Ki-67 expression in tumor tissues. **(F)** Representative gross images of lung and liver tissues from tail-vein injected mice (sh-NC vs. sh-ALYREF) and quantification of metastatic nodules. **(G)** HE staining of lung and liver tissues to evaluate metastatic lesions. **(H)** IHC staining and scoring of E-cadherin and Vimentin in metastatic lesions of the lung and liver. For tumor formation **(A–C)** and metastasis **(F)**, five mice per group were used; HE and IHC analyses were performed on three mice per group. Tumor growth curves **(B)** were analyzed by two-way repeated-measures ANOVA with Bonferroni *post hoc* test; other comparisons were made by two-tailed unpaired *t*-test. Data are presented as mean ± SD. *p < 0.05, **p < 0.01, ***p < 0.001.

To evaluate the role of ALYREF in metastasis, tail-vein injection models were used to mimic hematogenous dissemination. ALYREF silencing dramatically reduced lung metastatic nodule number and foci area (both *p* < 0.001), with no significant differences observed in the liver (Figures 3F,G). IHC analysis further demonstrated increased E-cadherin and decreased Vimentin levels in metastatic lesions following ALYREF knockdown (all *p* < 0.01), indicating suppression of EMT-mediated metastasis (Figure 3H).

### ALYREF drives glycolytic reprogramming in EC, with PKM2 identified as an m^5^C-modified target

Having established the oncogenic role of ALYREF in EC, we next sought to elucidate the underlying molecular mechanisms. Given that EC cells rely on glycolytic reprogramming to support aggressive phenotypes (Huang et al., 2023), we performed preranked GSEA of all 50 MSigDB Hallmark pathways in 545 unique primary TCGA-UCEC tumours. HALLMARK_GLYCOLYSIS was positively enriched among genes correlated with ALYREF (NES = 1.88; nominal *p* = 1.85 × 10^−6^; Benjamini–Hochberg-adjusted *p* = 7.13 × 10^−6^; 187 matched genes; Figure 4A), supporting an association between ALYREF expression and a glycolysis-related transcriptional programme. We next assessed whether ALYREF regulates glycolytic activity in EC cells. In Ishikawa cells, ALYREF knockdown significantly reduced glucose uptake, lactate production, and ATP generation. Conversely, ALYREF overexpression in HEC-1-A cells enhanced all three glycolytic indicators (all *p* < 0.05; Figures 4B–D), indicating that ALYREF facilitates glycolysis in EC.

**FIGURE 4:**
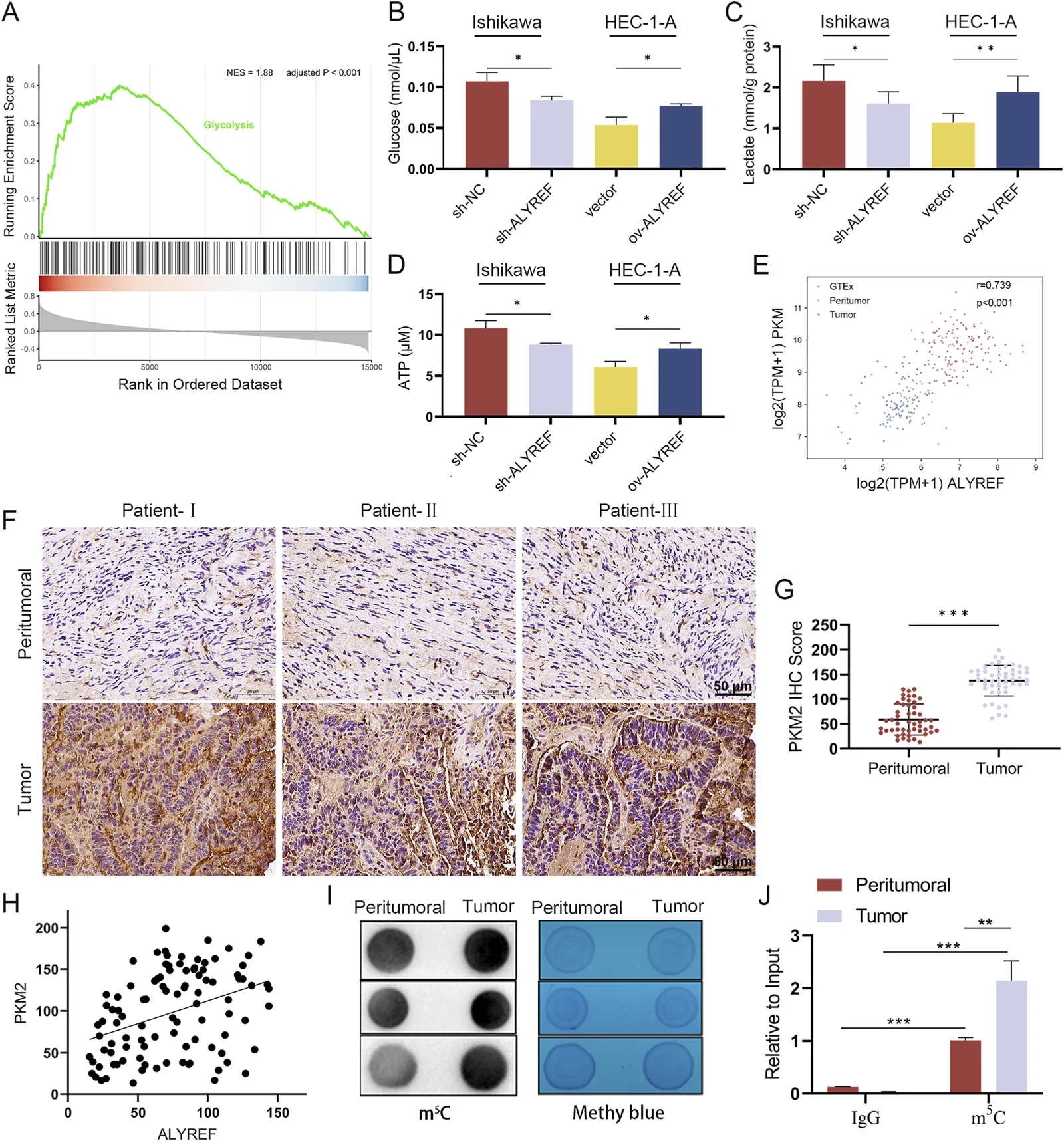
ALYREF promotes glycolysis in endometrial cancer cells by PKM2 regulation. **(A)** Preranked GSEA of HALLMARK_GLYCOLYSIS in 545 unique primary TCGA-UCEC tumours. A total of 14,867 unique protein-coding gene symbols were ranked by their Spearman correlation coefficients with ALYREF. The green curve shows the running enrichment score; black ticks show the positions of the 187 matched genes; the red-to-blue strip shows the positive-to-negative ranked correlation metric; and the grey distribution shows the ranked-list metric. HALLMARK_GLYCOLYSIS was positively enriched (NES = 1.88, nominal *p* = 1.85 × 10^−6^, Benjamini-Hochberg-adjusted *p* = 7.13 × 10^−6^). P values were adjusted across all 50 tested Hallmark pathways. **(B–D)** Glucose uptake **(B)**, lactate production **(C)**, and ATP levels **(D)** in Ishikawa and HEC-1-A cells with ALYREF knockdown or overexpression (three independent biological replicates). **(E)** GEPIA analysis showing the gene-level correlation between ALYREF and PKM across the selected UCEC tumour, UCEC normal, and GTEx uterus datasets (Spearman *r* = 0.739, *p* < 0.001). **(F,G)** Representative IHC staining of PKM2 in paired peritumoral and tumor tissues (50 pairs, images from three patients) **(F)** and quantification of IHC scores **(G)** (paired *t*-test). **(H)** Spearman correlation of ALYREF and PKM2 protein levels in 50 paired EC tissue samples. **(I)** Dot blot assay showing m^5^C modification levels in paired EC tissues from three independent patients. **(J)** m^5^C-RIP-qPCR analysis showing PKM2 mRNA enrichment in paired EC tissues from three independent patients. Data are shown as mean ± SD; *p < 0.05, **p < 0.01, ***p < 0.001.

Based on prior evidence that PKM2 is regulated by m^5^C-mediated mechanisms in cancer metabolism (Wang J. Z. et al., 2021), we examined PKM2 as a candidate downstream target. Initially, we performed GEPIA analysis to determine whether ALYREF expression correlates with PKM. This analysis revealed a positive gene-level association between ALYREF and PKM (Spearman *r* = 0.739, *p* < 0.001; Figure 4E). Building on this observation, we assessed PKM2 expression in paired EC tissues. RT-qPCR and western blot analyses consistently showed higher PKM2 expression in tumors compared with non-tumor controls (Supplementary Figure S1B). Expanding to a larger cohort, we performed IHC staining for PKM2 on the same 50 paired tissues. PKM2 was predominantly localized in the cytoplasm and was significantly upregulated in tumor tissues relative to adjacent normal tissues (*p* < 0.001; Figures 4F,G). Spearman correlation analysis confirmed a significant positive association between ALYREF and PKM2 protein levels in this cohort (*r* = 0.385, *p* < 0.001; Figure 4H). Since ALYREF functions as an m^5^C reader (Yang et al., 2017), we first examined global m^5^C levels in EC. Dot blot analysis of total RNA from three representative paired tissues showed globally elevated m^5^C levels in tumor tissues compared to adjacent tissues (Figure 4I). To directly test whether PKM2 mRNA carries m^5^C modifications, we performed m^5^C-IP followed by RT-qPCR on three representative paired tissues. PKM2 mRNA was significantly enriched by the m^5^C antibody in tumor versus normal tissues (*p* < 0.01; Figure 4J), confirming PKM2 as an m^5^C-modified target in EC.

### ALYREF stabilizes PKM2 mRNA to enhance glycolytic activity

Based on these findings, we next examined whether ALYREF regulates PKM2 post-transcriptionally. Western blot analyses showed that ALYREF knockdown reduced PKM2 protein levels (*p* < 0.001), whereas ALYREF overexpression induced PKM2 upregulation (*p* < 0.001; Figure 5A). Actinomycin D chase assays revealed that ALYREF prolongs the half-life of PKM2 mRNA, suggesting enhanced transcript stability (Figure 5B).

**FIGURE 5:**
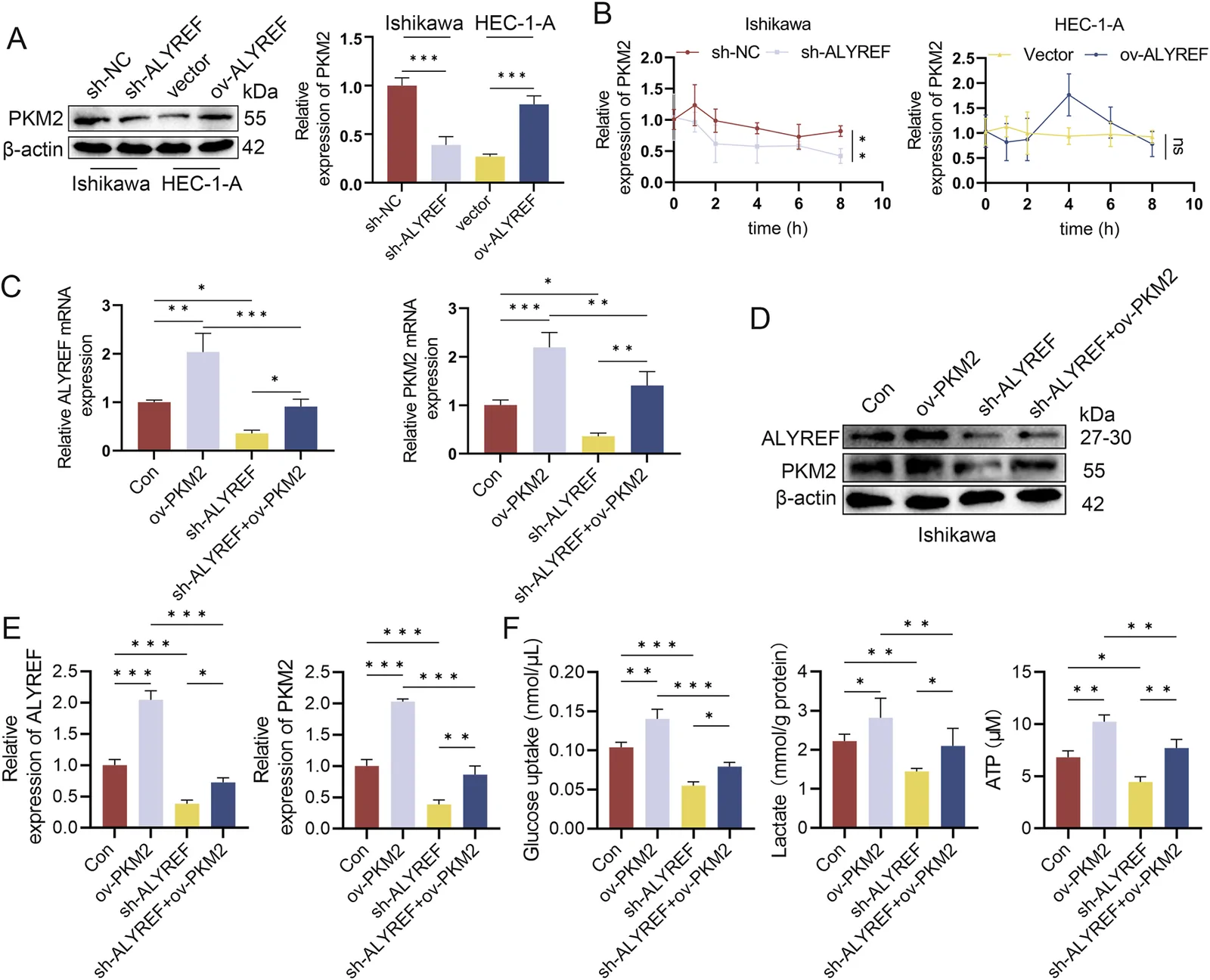
ALYREF stabilizes PKM2 mRNA to sustain glycolytic activity. **(A)** PKM2 expression levels in Ishikawa and HEC-1-A cells with or without ALYREF knockdown or overexpression. **(B)** Actinomycin D assay measuring PKM2 mRNA stability over time following ALYREF knockdown or overexpression. **(C)** Relative mRNA expression of PKM2 and ALYREF in the indicated groups (Con, ov-PKM2, sh-ALYREF, and sh-ALYREF + ov-PKM2) in Ishikawa cells, measured by quantitative real-time PCR (qRT-PCR). Values were normalized to β-actin. **(D,E)** Representative protein expression of PKM2 and ALYREF in Ishikawa cells analyzed by Western blot **(D)** and corresponding densitometric quantification **(E)**. β-Actin was used as a loading control. **(F)** Functional rescue assays measuring glucose uptake, lactate production, and ATP levels. All data are presented as mean ± SD from three independent biological replicates. **(A,B)** Two-tailed unpaired *t*-test **(C–F)** One-way ANOVA with Tukey’s *post hoc* test. *p < 0.05, **p < 0.01, ***p < 0.001.

Rescue experiments were then conducted to determine whether PKM2 mediates ALYREF-driven glycolysis. We first verified the molecular reconstitution of PKM2 in ALYREF-depleted cells. qRT-PCR and Western blot analyses confirmed that PKM2 overexpression effectively restored both PKM2 mRNA (Figure 5C) and protein levels (Figures 5D,E) in sh-ALYREF-treated Ishikawa cells. Functionally, PKM2 overexpression partially reversed the reduction in glycolytic indicators caused by ALYREF knockdown (glucose uptake and lactate production: *p* < 0.05; ATP: *p* < 0.01 vs. sh-ALYREF alone; Figure 5F), confirming that PKM2 contributes to ALYREF-dependent glycolytic activation.

### PKM2 contributes to the oncogenic effects of ALYREF in endometrial cancer cells

To determine whether PKM2 functions as the downstream effector of ALYREF in driving EC malignancy, we performed loss- and gain-of-function assays. We first assessed the intrinsic oncogenic role of PKM2 in Ishikawa cells. PKM2 knockdown efficiency was confirmed by Western blot (*p* < 0.01) and RT-qPCR (*p* < 0.001; Figure 6A). Subsequently, CCK-8 and colony formation assays were used to evaluate proliferative and clonogenic potential, whilst transwell systems with or without matrigel coating measured migratory and invasive capacity. PKM2 knockdown significantly suppressed cell viability (*p* < 0.01; Figure 6B), colony formation (*p* < 0.001; Figure 6C), migration (*p* < 0.001; Figure 6D), and invasion (*p* < 0.01; Figure 6E). To verify that ALYREF exerts its oncogenic effects through PKM2, we performed functional rescue assays by ectopically expressing PKM2 in ALYREF-silenced cells. PKM2 overexpression partially reversed the suppressive effects of ALYREF knockdown on proliferation, migration and invasion (Figures 6F–I). Finally, to investigate whether the ALYREF-PKM2 axis modulates the EMT programme that underpins enhanced tumour cell motility and invasion, we examined canonical EMT markers. ALYREF knockdown upregulated the epithelial marker E-cadherin and downregulated the mesenchymal markers N-cadherin, vimentin and matrix metalloproteinase MMP2; these molecular alterations were reversed by PKM2 overexpression (Figure 6J). Collectively, these findings demonstrate that PKM2 acts as an important mediator of ALYREF-driven tumor progression in EC.

**FIGURE 6:**
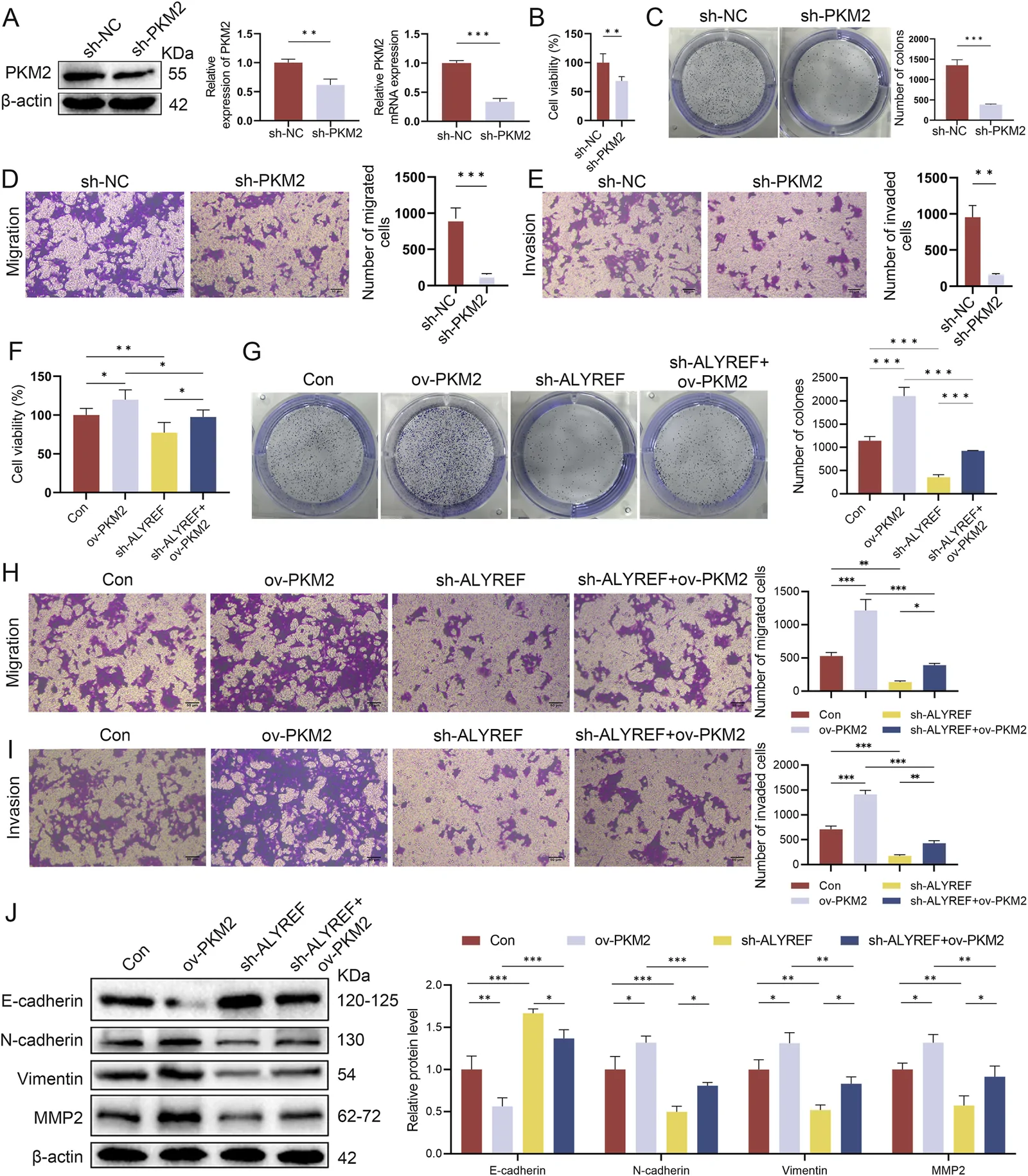
PKM2 contributes to the oncogenic effects of ALYREF in endometrial cancer cells **(A)** Validation of PKM2 knockdown efficiency at the protein and mRNA levels by Western blot and RT-qPCR, respectively. **(B,C)** Cell viability **(B)** and colony formation **(C)** assays demonstrating suppressed cell proliferation after PKM2 knockdown. **(D,E)** Transwell assays showing reduced cell migration **(D)** and invasion **(E)** abilities after PKM2 knockdown. **(F–I)** Functional rescue assays illustrating the effects of PKM2 overexpression on cell viability **(F)**, colony formation **(G)**, migration **(H)**, and invasion **(I)** in the indicated groups (Con, ov-PKM2, sh-ALYREF, and sh-ALYREF + ov-PKM2). **(J)** Western blot analysis and corresponding quantification of epithelial-mesenchymal transition (EMT) markers (E-cadherin, N-cadherin, Vimentin, and MMP2) across the indicated groups (Con, ov-PKM2, sh-ALYREF, and sh-ALYREF + ov-PKM2). β-actin was used as a loading control. All data are presented as mean ± SD from three independent biological replicates. **(A–E)** Two-tailed unpaired *t*-test; **(F–J)** One-way ANOVA with Tukey’s *post hoc* test. *p < 0.05, **p < 0.01, ***p < 0.001.

## Discussion

This study characterizes the oncogenic function of ALYREF in endometrial cancer (EC) and delineates an m^5^C-associated mechanism whereby ALYREF drives PKM2-mediated glycolytic reprogramming. We demonstrated that ALYREF is markedly upregulated in EC tissues and cell lines. In IHC analysis of 50 paired EC specimens, high ALYREF expression was significantly associated with advanced FIGO stage, higher histological grade, deeper myometrial invasion, lymph node metastasis, and shorter survival (<5 years). A gene-level association between ALYREF and PKM was observed in the selected GEPIA datasets, whereas a positive association between ALYREF and PKM2 protein levels was confirmed in our clinical cohort. Functionally, ALYREF promoted EC cell proliferation, migration, invasion and EMT *in vitro*, and accelerated tumor growth and metastasis *in vivo*. Mechanistically, ALYREF enhanced PKM2 mRNA stability and increased PKM2 protein levels, thereby activating glycolytic flux. These findings identify ALYREF as a critical epitranscriptomic regulator that links RNA modification with metabolic reprogramming in endometrial cancer.

As a canonical m^5^C “reader” protein, ALYREF was originally identified as a cofactor involved in mRNA export and processing (Pacheco-Fiallos et al., 2023). Beyond its nuclear export role, ALYREF can stabilize m^5^C-modified transcripts in an m^5^C-dependent manner (Wang et al., 2023) and maintain genome stability by resolving R-loops (Bhandari et al., 2024). Several studies have reported that RNA chemical modifications such as m^5^C and m^6^A can regulate tumor metabolic adaptation by modulating the stability and translational efficiency of mRNAs encoding metabolic enzymes and signaling components (Shi et al., 2026; Qing et al., 2023). Recent studies have further suggested that ALYREF participates in metabolic gene regulation, such as controlling the proliferation, differentiation, and glycolysis of antler chondrocytes through m^5^C-dependent circRNA modification (Yao et al., 2025). In line with this trend, ALYREF has also been implicated in metabolic gene regulation in cancer. Previous studies have described a pro-tumorigenic ALYREF-PKM2 axis in bladder cancer (Wang J. Z. et al., 2021) and intrahepatic cholangiocarcinoma (Wang et al., 2025), where it drives glucose metabolism and tumour progression. However, whether this axis operates in EC, a hormonally driven gynaecological malignancy with distinct metabolic features, remained poorly explored. Our results demonstrate that ALYREF upregulates PKM2 expression in an m^5^C-associated manner, thereby driving glycolytic reprogramming in EC. This mechanism is supported by our finding that PKM2 mRNA carries m^5^C modifications and that ALYREF depletion shortens its half-life, indicating that ALYREF stabilizes this transcript and thereby contributes to PKM2 upregulation. However, the m^5^C methyltransferase responsible for PKM2 mRNA modification in EC remains unidentified in this study. It has been reported that NSUN2, an m^5^C writer, is upregulated and exerts oncogenic functions in EC (Chen et al., 2024). In other cancer types, prior work has shown that NSUN2 mediates m^5^C methylation of PKM2 mRNA and contributes to its ALYREF-dependent stabilisation (Wang J. Z. et al., 2021; Wang et al., 2025). Whether NSUN2 acts as the upstream methyltransferase for the ALYREF-PKM2 regulatory pathway in EC requires further investigation, and involvement of additional m^5^C methyltransferases cannot be formally excluded.

PKM2 is a rate-limiting enzyme of glycolysis, catalyzing the terminal step of the glycolytic pathway. Beyond its cytoplasmic metabolic function, PKM2 also acts as a nuclear coactivator that regulates transcription factors such as HIF-1α and STAT3 (Taiyab et al., 2025; Chen et al., 2025). It is frequently overexpressed in various cancers and correlates with poor prognosis (Lai et al., 2019). In EC, we similarly observed PKM2 upregulation, and PKM2 knockdown impaired EC cell proliferation, migration and invasion. Notably, PKM2 expression positively correlated with ALYREF in our cohort. Moreover, reintroduction of PKM2 partially restored proliferative, migratory and invasive capacities in ALYREF-depleted cells, indicating that PKM2 acts downstream of ALYREF to drive these malignant phenotypes. These findings establish a functional link between ALYREF and PKM2-driven glycolytic reprogramming in EC.

The ALYREF-PKM2 axis appears to coordinate both metabolic and phenotypic programs in EC. In our study, ALYREF silencing not only reduced glycolytic output, as evidenced by decreased glucose uptake, lactate production and ATP levels, but also suppressed EMT marker alterations (E-cadherin upregulation alongside N-cadherin and Vimentin downregulation) and impaired migratory and invasive capacities *in vitro*, which translated to diminished distant metastasis *in vivo*. Reintroduction of PKM2 partially rescued these defects, indicating that PKM2 contributes to ALYREF-driven malignant phenotypes. Increasing evidence supports reciprocal crosstalk between metabolic reprogramming and epigenetic dysregulation in cancer (Shi et al., 2026; Agnihotri and Zadeh, 2016). Against this backdrop, our findings position the ALYREF-PKM2 axis as a functional link between m^5^C-associated RNA stabilisation and metabolic reprogramming in EC. Future multi-omics studies integrating m^5^C mapping and metabolomic profiling will be valuable to delineate the broader downstream landscape of ALYREF-regulated metabolic pathways beyond the PKM2-driven glycolysis axis.

From a translational perspective, ALYREF is broadly overexpressed across multiple malignancies and has been linked to radio- and chemoresistance as well as poor prognosis (Yuan et al., 2024). Given the reversibility of RNA methylation, targeting ALYREF or its m^5^C-binding interface may represent a therapeutic strategy. PKM2 is highly expressed in EC (Jin et al., 2025) and has been proposed as a biomarker for identifying high-risk patients and guiding therapeutic decisions (Teng et al., 2013). In agreement with findings in intrahepatic cholangiocarcinoma, where the ETV4/ALYREF–PKM2 metabolic axis promotes tumor progression (Wang et al., 2025), our data support a similar ALYREF-driven PKM2 upregulation in EC, suggesting that m^5^C-mediated regulation or PKM2 inhibition could represent a potential metabolic anti-tumor approach (Zhang et al., 2019; Zhu et al., 2021; Wang D. et al., 2021). Moreover, several natural and synthetic PKM2 modulators have been developed (Su et al., 2019); PKM2 inhibitors have shown antiproliferative activity in cervical and breast cancer models (Rivera-Ávalos et al., 2019), but their therapeutic efficacy in EC remains to be validated.

Despite these advances, our study has several limitations. First, although PKM2 mRNA is m^5^C-modified in EC and its stability is regulated by ALYREF, direct binding of ALYREF to PKM2 mRNA and the exact m^5^C residue(s) were not identified in this study. Future work using ALYREF RIP, m^5^C site mapping, and mutant reporter assays will clarify the precise mechanism. Second, although rescue experiments confirmed PKM2 as an important downstream mediator of ALYREF, re-expression of PKM2 only partially restored the malignant phenotypes, suggesting that additional ALYREF-regulated effectors contribute to its oncogenic activity; genome-wide m^5^C profiling is needed to define these targets. Third, the clinical sample size in our study was limited, and multi-center cohorts are required to confirm the prognostic significance of ALYREF. Fourth, the upstream regulatory mechanisms governing ALYREF expression, such as hormonal, oxidative, or metabolic cues, remain to be elucidated. Future studies combining single-cell m^5^C sequencing, spatial metabolomics, and flux analysis will help reveal how the ALYREF-m^5^C network reshapes the tumor microenvironment and metabolic plasticity in endometrial cancer. Finally, although our findings suggest that ALYREF and PKM2 may be potential therapeutic targets, no pharmacological inhibitors were tested in this study. The therapeutic implications of the ALYREF-PKM2 axis are therefore prospective and require validation in future preclinical studies.

## Conclusion

In summary, this study delineates a mechanism by which the RNA-binding protein ALYREF promotes endometrial cancer progression through m^5^C-associated stabilization of PKM2 mRNA. ALYREF enhances PKM2 expression, drives glycolytic reprogramming, and facilitates tumor cell proliferation, migration, and invasion. Functional rescue experiments confirmed PKM2 as an important downstream effector of ALYREF-mediated oncogenic activity. Collectively, our findings provide mechanistic insights into the interplay between RNA methylation and metabolic regulation, suggesting that the ALYREF/m^5^C/PKM2 axis may serve as a potential therapeutic target for EC.

## Data Availability

Public datasets analysed in this study were obtained from public repositories. Raw uncropped Western blot membranes are included in the Supplementary Material. Other underlying experimental data are available from the corresponding author upon reasonable request.

## References

[B1] Agnihotri S. Zadeh G. (2016). Metabolic reprogramming in glioblastoma: the influence of cancer metabolism on epigenetics and unanswered questions. Neuro Oncol. 18, 160–172. 10.1093/neuonc/nov125 26180081 PMC4724176

[B2] Aiello N. M. Kang Y. (2019). Context-dependent EMT programs in cancer metastasis. J. Exp. Med. 216, 1016–1026. 10.1084/jem.20181827 30975895 PMC6504222

[B3] Bhandari J. Guillén-Mendoza C. Banks K. Eliaz L. Southwell S. Eyaa D. (2024). The molecular chaperone ALYREF promotes R-loop resolution and maintains genome stability. J. Biol. Chem. 300, 107996. 10.1016/j.jbc.2024.107996 39547511 PMC11665464

[B4] Chen X. Li A. Sun B. F. Yang Y. Han Y. N. Yuan X. (2019). 5-methylcytosine promotes pathogenesis of bladder cancer through stabilizing mRNAs. Nat. Cell Biol. 21, 978–990. 10.1038/s41556-019-0361-y 31358969

[B5] Chen S. J. Zhang J. Zhou T. Rao S. S. Li Q. Xiao L. Y. (2024). Epigenetically upregulated NSUN2 confers ferroptosis resistance in endometrial cancer via m(5)C modification of SLC7A11 mRNA. Redox Biol. 69, 102975. 10.1016/j.redox.2023.102975 38042059 PMC10711489

[B6] Chen T. J. Wu C. H. Hung M. C. Wang W. C. Kung H. J. (2025). Nuclear PKM2: a signal receiver, a gene programmer, and a metabolic modulator. J. Biomed. Sci. 32, 75. 10.1186/s12929-025-01170-6 40790192 PMC12341297

[B7] Corr B. R. Erickson B. K. Barber E. L. Fisher C. M. Slomovitz B. (2025). Advances in the management of endometrial cancer. Bmj 388, e080978. 10.1136/bmj-2024-080978 40044230

[B8] Crosbie E. J. Kitson S. J. McAlpine J. N. Mukhopadhyay A. Powell M. E. Singh N. (2022). Endometrial cancer. Lancet 399, 1412–1428. 10.1016/s0140-6736(22)00323-3 35397864

[B9] He W. Hong X. Chen G. Luo X. Lin Y. (2024). RNA modifications in gynecological cancer: current status and future directions. Front. Med. (Lausanne) 11, 1314075. 10.3389/fmed.2024.1314075 38343637 PMC10853395

[B10] Huang P. Fan X. Yu H. Zhang K. Li H. Wang Y. (2023). Glucose metabolic reprogramming and its therapeutic potential in obesity-associated endometrial cancer. J. Transl. Med. 21, 94. 10.1186/s12967-022-03851-4 PMC9906873 36750868

[B11] Jin Y. Yao J. Fu J. Huang Q. Luo Y. You Y. (2024). ALYREF promotes the metastasis of nasopharyngeal carcinoma by increasing the stability of NOTCH1 mRNA. Cell Death Dis. 15, 578. 10.1038/s41419-024-06959-1 PMC11310353 39117671

[B12] Jin Y. Qi M. Si L. Shi X. Cai M. Fu H. (2025). IGFBP2 promotes proliferation and glycolysis of endometrial cancer by regulating PKM2/HIF-1α axis. Cancer Sci. 116, 656–672. 10.1111/cas.16447 39761954 PMC11875784

[B13] Khoddami V. Cairns B. R. (2013). Identification of direct targets and modified bases of RNA cytosine methyltransferases. Nat. Biotechnol. 31, 458–464. 10.1038/nbt.2566 23604283 PMC3791587

[B14] Lai Y. J. Chou Y. C. Lin Y. J. Yu M. H. Ou Y. C. Chu P. W. (2019). Pyruvate kinase M2 expression: a potential metabolic biomarker to differentiate endometrial precancer and cancer That Is associated with poor outcomes in endometrial carcinoma. Int. J. Environ. Res. Public Health 16, 4589. 10.3390/ijerph16234589 31756939 PMC6926947

[B15] Lu Y. Yang L. Feng Q. Liu Y. Sun X. Liu D. (2024). RNA 5-Methylcytosine modification: regulatory molecules, biological functions, and human diseases. Genomics Proteomics Bioinforma. 22, qzae063. 10.1093/gpbjnl/qzae063 39340806 PMC11634542

[B16] Markatos C. Biniari G. Karageorgos V. Chepurny O. G. Venihaki M. Holz G. G. (2024). Two GnRH-mitoxantrone conjugates, Con-3 and Con-7, target endometrial cancer cells. Curr. Mol. Pharmacol. 17, e18761429343090. 10.2174/0118761429343090250121052955 39902544 PMC13735368

[B17] Miao L. Chen B. Li L. Ma B. Yang G. Jing L. (2025). Role of TPD52 in endometrial cancer: impact on EMT and the PI3K/AKT and ERK/MAPK signaling. Protein Pept. Lett. 32, 718–730. 10.2174/0109298665400400251003013958 41140081

[B18] Pacheco-Fiallos B. Vorländer M. K. Riabov-Bassat D. Fin L. O'Reilly F. J. Ayala F. I. (2023). mRNA recognition and packaging by the human transcription-export complex. Nature 616, 828–835. 10.1038/s41586-023-05904-0 37020021 PMC7614608

[B19] Qing Y. Wu D. Deng X. Chen J. Su R. (2023). RNA modifications in cancer metabolism and tumor microenvironment. Cancer Treat. Res. 190, 3–24. 10.1007/978-3-031-45654-1_1 38112997

[B20] Rivera-Ávalos E. de Loera D. Araujo-Huitrado J. G. Escalante-García I. L. Muñoz-Sánchez M. A. Hernández H. (2019). Synthesis of amino acid-naphthoquinones and *in vitro* studies on cervical and breast cell lines. Molecules 24, 4285. 10.3390/molecules24234285 31775253 PMC6930466

[B21] Roundtree I. A. Evans M. E. Pan T. He C. (2017). Dynamic RNA modifications in gene expression regulation. Cell 169, 1187–1200. 10.1016/j.cell.2017.05.045 28622506 PMC5657247

[B22] Shi Q. Liu Y. Xuan M. Fu L. Lou N. Lv C. (2026). Recent advances in m6A RNA modification in hepatocellular carcinoma: from mechanisms to therapeutic potential. Front. Mol. Biosci. 13, 1772310. 10.3389/fmolb.2026.1772310 PMC13046554 41938016

[B23] Squires J. E. Patel H. R. Nousch M. Sibbritt T. Humphreys D. T. Parker B. J. (2012). Widespread occurrence of 5-methylcytosine in human coding and non-coding RNA. Nucleic Acids Res. 40, 5023–5033. 10.1093/nar/gks144 22344696 PMC3367185

[B24] Su Q. Luo S. Tan Q. Deng J. Zhou S. Peng M. (2019). The role of pyruvate kinase M2 in anticancer therapeutic treatments. Oncol. Lett. 18, 5663–5672. 10.3892/ol.2019.10948 31788038 PMC6865080

[B25] Sun Z. Xue S. Zhang M. Xu H. Hu X. Chen S. (2020). Aberrant NSUN2-mediated m(5)C modification of H19 lncRNA is associated with poor differentiation of hepatocellular carcinoma. Oncogene 39, 6906–6919. 10.1038/s41388-020-01475-w 32978516 PMC7644462

[B26] Taiyab A. Hasan G. M. Zulfareen Hassan M. I. (2025). A review on the role of pyruvate kinase M2 in cancer: from metabolic switch to transcriptional regulation. Int. J. Biol. Macromol. 330, 148067. 10.1016/j.ijbiomac.2025.148067 41046087

[B27] Teng Y. Ai Z. Wang Y. Wang J. Luo L. (2013). Proteomic identification of PKM2 and HSPA5 as potential biomarkers for predicting high-risk endometrial carcinoma. J. Obstet. Gynaecol. Res. 39, 317–325. 10.1111/j.1447-0756.2012.01970.x 22889453

[B28] Tian Y. Liu J. Sun L. Wang X. (2025). ALYREF regulates the m5C modification and stability of BIRC5 mRNA to promote ovarian cancer progression. Pathol. Res. Pract. 272, 156055. 10.1016/j.prp.2025.156055 40479805

[B29] Wang J. Z. Zhu W. Han J. Yang X. Zhou R. Lu H. C. (2021). The role of the HIF-1α/ALYREF/PKM2 axis in glycolysis and tumorigenesis of bladder cancer. Cancer Commun. (Lond) 41, 560–575. 10.1002/cac2.12158 33991457 PMC8286140

[B30] Wang D. Zhao C. Xu F. Zhang A. Jin M. Zhang K. (2021). Cisplatin-resistant NSCLC cells induced by hypoxia transmit resistance to sensitive cells through exosomal PKM2. Theranostics 11, 2860–2875. 10.7150/thno.51797 33456577 PMC7806469

[B31] Wang N. Chen R. X. Deng M. H. Wei W. S. Zhou Z. H. Ning K. (2023). m(5)C-dependent cross-regulation between nuclear reader ALYREF and writer NSUN2 promotes urothelial bladder cancer malignancy through facilitating RABL6/TK1 mRNAs splicing and stabilization. Cell Death Dis. 14, 139. 10.1038/s41419-023-05661-y 36806253 PMC9938871

[B32] Wang X. Duan W. Ma Z. Wen H. Mao X. Liu C. (2025). ETV4/ALYREF-mediated glycolytic metabolism through PKM2 enhances resistance to ferroptosis and promotes the development of intrahepatic cholangiocarcinoma. Cancer Metab. 13, 19. 10.1186/s40170-025-00387-1 PMC12013154 40264211

[B33] Xie Q. Chen L. Huang Y. Yu Z. Zhu R. Li J. (2025). Autophagy-dependent secretion of ENO1 mediates chemoresistance of glioblastoma and tumor microenvironment remodeling. Cell Death Dis. 17, 79. 10.1038/s41419-025-08313-5 41353343 PMC12827997

[B34] Xue C. Gu X. Zheng Q. Shi Q. Yuan X. Su Y. (2023). ALYREF mediates RNA m(5)C modification to promote hepatocellular carcinoma progression. Signal Transduct. Target Ther. 8, 130. 10.1038/s41392-023-01395-7 36934090 PMC10024699

[B35] Yang X. Yang Y. Sun B. F. Chen Y. S. Xu J. W. Lai W. Y. (2017). 5-methylcytosine promotes mRNA export - NSUN2 as the methyltransferase and ALYREF as an m(5)C reader. Cell Res. 27, 606–625. 10.1038/cr.2017.55 28418038 PMC5594206

[B36] Yang S. Luo Y. Zhou D. Xiang J. Xi X. (2023). RNA 5-Methylcytosine regulators are associated with cell adhesion and predict prognosis of endometrial cancer. Transl. Cancer Res. 12, 2556–2571. 10.21037/tcr-23-742 37969377 PMC10643971

[B37] Yang Q. Salih S. M. Wu R. Arora I. Mousa M. Al-Hendy A. (2026). 5-Methylcytidine RNA epitranscriptomics in women's health and disease: mechanisms and clinical implications. Cells 15, 847. 10.3390/cells15090847 PMC13162702 42121948

[B38] Yao H. Song M. Zhang H. Li Y. Chen D. Li Y. (2025). NSUN2/ALYREF-medicated m5C-modified circRNA505 regulates the proliferation, differentiation, and glycolysis of antler chondrocytes via the miRNA-127/p53 axis and LDHA. Int. J. Biol. Macromol. 309, 142527. 10.1016/j.ijbiomac.2025.142527 40147663

[B39] Ye J. Wen X. Liu R. Huang C. Xu Z. Zhan Y. (2026). ZCCHC4 orchestrates hepatocellular carcinoma metastasis by regulating lipid biosynthesis and TMEM97/LCN2/Twist1 pathway. Int. J. Biol. Sci. 22, 3600–3616. 10.7150/ijbs.120086 42003930 PMC13086013

[B40] Yuan Y. Fan Y. Tang W. Sun H. Sun J. Su H. (2024). Identification of ALYREF in pan cancer as a novel cancer prognostic biomarker and potential regulatory mechanism in gastric cancer. Sci. Rep. 14, 6270. 10.1038/s41598-024-56895-5 38491127 PMC10942997

[B41] Zhang Z. Deng X. Liu Y. Liu Y. Sun L. Chen F. (2019). PKM2, function and expression and regulation. Cell Biosci. 9, 52. 10.1186/s13578-019-0317-8 31391918 PMC6595688

[B42] Zhang L. Li Y. Li L. Yao F. Cai M. Ye D. (2025). Detection, molecular function and mechanisms of m5C in cancer. Clin. Transl. Med. 15, e70239. 10.1002/ctm2.70239 PMC11862898 40008496

[B43] Zhu S. Guo Y. Zhang X. Liu H. Yin M. Chen X. (2021). Pyruvate kinase M2 (PKM2) in cancer and cancer therapeutics. Cancer Lett. 503, 240–248. 10.1016/j.canlet.2020.11.018 33246091

