# Supplementary material for: ALYREF regulates glycolysis and promotes the progression of endometrial cancer through m5C modification of PKM2 mRNA
Source: Front Cell Dev Biol. 2026 Oct 2;14:1831562. doi: 10.3389/fcell.2026.1831562 (PMC13672539; doi:10.3389/fcell.2026.1831562)
Supplement: Supplementary file 1 [file Supplementaryfile1.docx]

**Supplementary Figure S1. Validation of ALYREF and PKM2 expression in paired EC tissues.** (A) Western blot and quantification of ALYREF protein levels. (B) RT-qPCR and Western blot analysis of PKM2 expression. Paired EC tumor and adjacent normal tissues from three independent patients were analyzed as biological replicates. Each matched pair (Tumor 1/Peritumoral 1, Tumor 2/Peritumoral 2, Tumor 3/Peritumoral 3) was derived from the same patient. β-actin served as the loading control. Data are presented as mean ± SD; **p* < 0.05, ***p* < 0.01, ****p* < 0.001 by paired *t*-test.

Supplementary Table S1. shRNA sequences used for ALYREF knockdown.

| shRNA Name | Target Gene | Target Sequence (5'-3') | Purpose |
| --- | --- | --- | --- |
| sh-ALYREF | ALYREF | GCCGATATTCAGGAACTCTTT | knockdown |
| sh-NC | None (scrambled) | CCTAAGGTTAAGTCGCCCTCG | Negative control |
